# Incidence Trend and Competing Risk Analysis of Patients With Intrahepatic Cholangiocarcinoma: A Population-Based Study

**DOI:** 10.3389/fmed.2022.846276

**Published:** 2022-03-30

**Authors:** Huiwu Xing, Bingqian Tan, Chenyu Yang, Mingman Zhang

**Affiliations:** Department of Hepatobiliary Surgery, Children’s Hospital of Chongqing Medical University, Chongqing Key Laboratory of Pediatrics, National Clinical Research Center for Child Health and Disorders, Ministry of Education Key Laboratory of Child Development and Disorders, Chongqing, China

**Keywords:** intrahepatic cholangiocarcinoma, prognosis, risk factor, nomogram, liver transplantation

## Abstract

**Background:**

Intrahepatic cholangiocarcinoma (ICCA) is a primary liver cancer characterized by rapid progression and poor prognosis. There are few effective tools for evaluating the prognosis of ICCA patients, and the use of liver transplantation (LT) of the treatment for ICCA is still controversial.

**Methods:**

We analyzed ICCA incidence data and clinicopathological data from the Surveillance, Epidemiology, and End Results database. Prognostic predictors were identified by univariate and multivariate *Cox* regression analyses and then used to establish a nomogram. The prediction performance of the nomogram was evaluated with receiver operating characteristic (ROC) curves, calibration plots and decision curve analysis (DCA) plots. Propensity score matching (PSM) was used to balance the baseline data of patients undergoing LT and other operations, and then, univariate *Cox* regression analysis was used to evaluate the therapeutic value of LT for ICCA.

**Results:**

The incidence of ICCA increased significantly, from 0.6 per 100,000 in 2,000 to 1.3 per 100,000 in 2018. The median overall survival (OS) of the patients was 13 months, and the 1-, 3-, and 5-year OS rates were 51.40, 22.14, and 13.79%, respectively. *Cox* regression analysis showed that age under 60 years old, female, tumor size ≤ 50 mm, better differentiation, smaller range of tumor invasion, lack of distant metastasis, regional lymph node surgery and treatment were associated with a better prognosis. The ROC curves, calibration plots, and DCA plots showed that the nomogram had good discrimination and calibration power, as well as clinical utility. After PSM, the univariate *Cox* regression analysis showed no significant difference in OS between patients treated with LT and patients treated with other operations.

**Conclusion:**

The incidence of ICCA increased significantly. A nomogram with good predictive performance was developed to predict the OS of ICCA patients. LT might be considered as a potential option for some ICCA patients.

## Introduction

Intrahepatic cholangiocarcinoma (ICCA) is one of the most prevalent types of primary liver cancer, and its prevalence is second only to that of hepatocellular carcinoma (HCC) (1). ICCA originates from the branch bile duct above the left or right bile duct (secondary bile duct); it is exceptionally aggressive and has a poor prognosis. ICCA is very different from the other two anatomic subtypes of cholangiocarcinoma in terms of symptoms, imaging features, treatment, and prognosis (2, 3). Studies have reported that the incidence of ICCA has increased year over year (4, 5). The rates of mortality due to extrahepatic cholangiocarcinoma have decreased in most countries, but those due to ICCA showed an increasing trend worldwide (6). In addition, the number of papers about ICCA indexed yearly by PubMed increased exponentially from 1950 to 2021. These findings suggest that ICCA has increasingly become a public health problem.

Almost all guidelines recommend that surgical resection is the only potentially curative treatment for ICCA, especially for resectable lesions (7–9). However, ICCA is prone to recur, and the prognosis is poor because of the difficulty in achieving complete resection (10, 11) and the prevalence of risk factors, including viral or parasitic infections, hepatolithiasis, primary sclerosing cholangitis (PSC), and metabolic diseases (6, 9). Liver transplantation (LT) seems to be a good choice for treating ICCA patients with multiple lesions, inadequate future liver remnant (FLR) or insufficient liver function reserve, and LT could eliminate poor prognostic factors such as liver cirrhosis and other occult lesions (12, 13). Nevertheless, due to donor shortages, high recurrence rates and low substandard survival rates, whether LT should be used to treat ICCA is still controversial (14, 15).

In the current era of individualized medicine, more effective tools for predicting disease prognosis are beneficial for decreasing excessive intervention and improving doctor–patient communication. Compared with conventional staging methods, nomograms are more individualized and easy to understand (16). However, sample size and source can affect the accuracy of the nomogram. The Surveillance, Epidemiology, and End Results (SEER) database^1^ has the advantages of including data across a long-time span and from a large population, and this database is beneficial for medical research on rare diseases.

In this study, we analyzed the clinicopathologic variables and incidence data of ICCA based on data from the SEER database, further studied the incidence trend of ICCA, and established a nomogram related to the prognosis of patients with ICCA. We also evaluated the value of LT in the treatment of ICCA after propensity score matching (PSM).

## Materials and Methods

### Patients and Study Design

**FIGURE 1:**
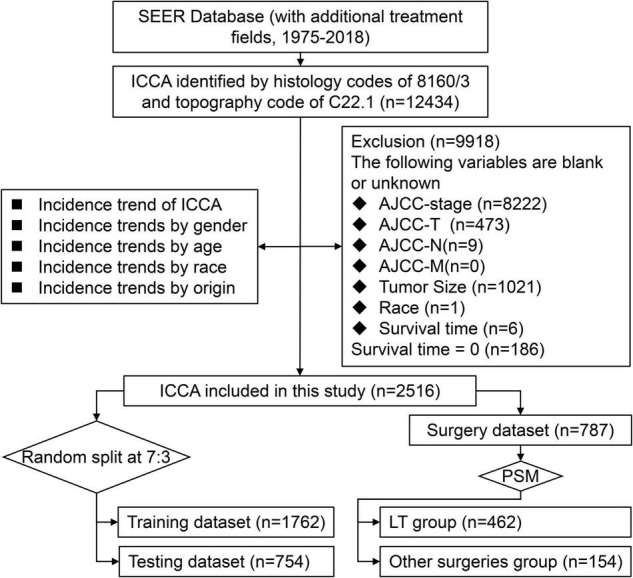
Flowchart of screening patients with ICCA in the SEER database.

For this retrospective population-based study, we obtained incidence data from 2000 to 2018 and clinicopathological data from 1975 to 2018 from the Rate and Case listing sessions from the SEER database with SEER*Stat software (version 8.3.9).^2^ Patients with a histology code of 8,160/3 and a topography code of C22.1 were enrolled in this study (*n* = 12,434). Because the AJCC staging system (8th edition) was not used in the SEER database at the time of this study, the AJCC staging system (7th edition) was used. Patients with any of the following variables missing were excluded: staging groups (AJCC stage), primary tumor (AJCC-T), regional lymph nodes (AJCC-N), distant metastasis (AJCC-M), tumor size, race, and survival time (*n* = 9,918). Then, patients with survival time of less than 1 month were excluded (*n* = 186). The included patients were randomly divided into the training dataset and testing dataset at a ratio of 7:3, and patients who underwent surgery were included in the surgery dataset. We used the training dataset to analyze the prognosis of ICCA and establish the nomogram, and then used the testing dataset to verify the effectiveness of the nomogram (17). We used the surgery dataset including patients underwent segmental resection, lobectomy, hepatectomy and LT to evaluate the therapeutic value of LT for ICCA (Figure 1).

### Variable Definitions

The predictive covariates were categorized as demographic, and clinical variables. Demographic variables included age of diagnosis, race, origin, and marital status at diagnosis. Clinical-pathological variables included grade, tumor size, AJCC stage, AJCC-T, AJCC-N, AJCC-M, regional lymph node (LN) surgery, radiotherapy, and chemotherapy. Patients were classified into three age groups: < 60, 60–79, and = 80 years old. The histological grades included I (well differentiated), II (moderately differentiated), III (poorly differentiated) and IV (undifferentiated). Since all cases had been diagnosed and included to SEER database until 2018, it was not possible to subclassify cases as: large duct/small duct/cholangiocarcinoma, as more recently defined by WHO Classification of Tumors—5th Edition, Digestive System Tumors, 2019. Adjuvant therapy included preoperative or postoperative radiotherapy alone, chemotherapy alone or a combination of radiotherapy and chemotherapy. Systemic therapy was defined as surgery combined with adjuvant therapy. The survival status was defined as alive or death due to all causes, and the survival time was the overall survival (OS) in months which was defined as the interval measured between diagnosis and death or last follow-up.

### Statistical Analysis

The age-adjusted incidence trend of ICCA was calculated according to the 2,000 US standard population. The annual percentage change (APC) values were calculated using the *weighted least squares* method. The APC is significantly different from zero (*p* < 0.05). We plotted incidence trends and performed linear regression using the *ggplot2* package in *R* software (version 4.0.2).^3^

For statistical comparison of the baseline characteristics between groups, the *chi-square-test* or *Fisher’s exact* test was used for categorical variables. Survival curves were drawn by the *Kaplan-Meier method*, and these curves were used to calculate the 1-, 3-, and 5-year OS rates. The impact of variables on prognosis was evaluated by univariate and multivariate *Cox* regression analyses of the training dataset. Candidate variables with two-tailed *p* < 0.05 in the univariate *Cox* regression analysis were included in the multivariable *Cox* regression analysis, and variables with two-tailed *p* < 0.05 in the multivariate *Cox* regression analysis were defined as independent factors, which were used to establish a nomogram. We verified the nomogram internally using the testing dataset. We used the area under the curve (AUC) of receiver operating characteristic (ROC) curves to evaluate the discrimination, calibration plots to evaluate the calibration, and DCA plots to evaluate the clinical utility of the nomogram (16). The distribution of the LT group and other operations group in the surgery dataset was not random, and unbalanced characteristics may lead to selection bias, thus affecting the accuracy of the analysis results. Therefore, we used PSM to minimize but not eliminate selection bias to create a highly comparable dataset. For PSM, we considered the following covariates: age at diagnosis, race, gender, AJCC-T, AJCC-N, and AJCC-M. Then, the LT group was matched to the other operation group based on the calculated scores with an algorithm of nearest neighbor 1:3 matching (18). We used univariate *Cox* regression to compare the difference in prognosis between the LT and other operation groups. Two-tailed *p* < 0.05 was considered statistically significant. All the analyses were performed using the *caret*, *survival*, *survminer*, *tableone*, *rms*, *timeROC*, *pec*, *foreign* and *MatchIt* packages in *R* software.

## Results

### Incidence Trends of Intrahepatic Cholangiocarcinoma

**FIGURE 2:**
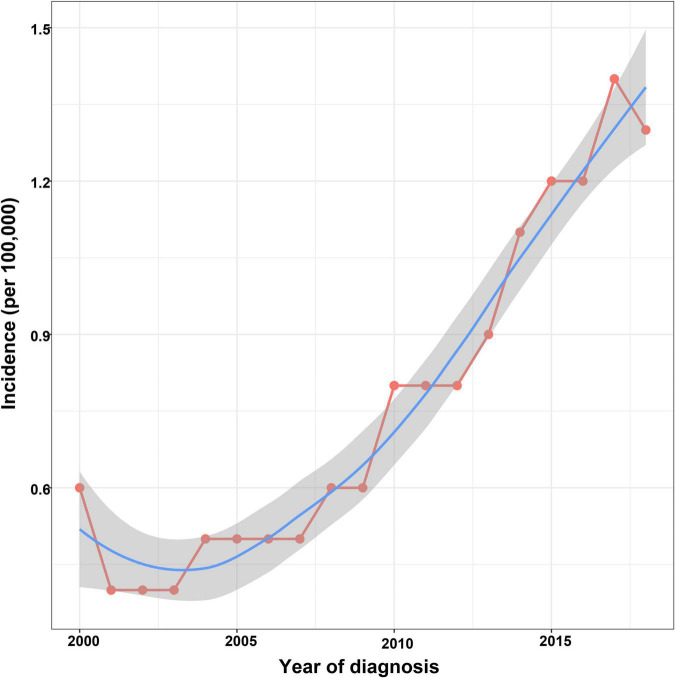
Incidence trend of patients with ICCA.

**FIGURE 3:**
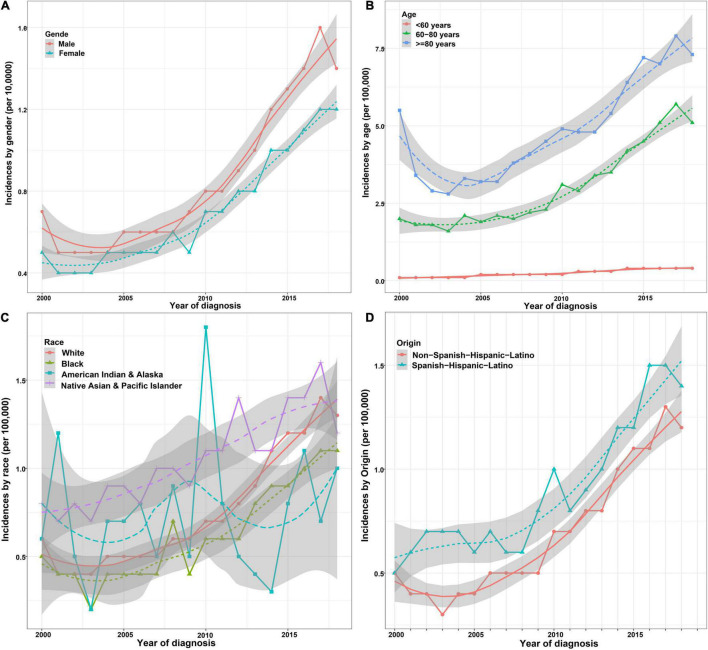
Incidence trends of patients with ICCA in different demographic groups. **(A)** Incidence trends of patients with ICCA between different gender groups. **(B)** Incidence trends of patients with ICCA between different age groups. **(C)** Incidence trends of patients with ICCA between different race groups. **(D)** Incidence trends of patients with ICCA between different origin groups.

The incidence of ICCA increased significantly from 0.6 per 100,000 in 2,000 to 1.3 per 100,000 in 2018, with an APC of 7.3% (95% CI: 6.2–8.4%) (Figure 2). Additionally, we found that the incidences of ICCA in all gender and origin groups increased significantly (Figures 3A,D). Moreover, the incidences of ICCA in 60–80 and ≥ 80 year-old groups increased, whereas that in < 60 year-old group remained low and relatively stable (from 0.1 per 100,000 in 2,000 to 0.4 per 100,000 in 2018) (Figure 3B). Among the different racial groups, the incidence of ICCA in the white group increased more significantly (Figures 3C,D).

### Patient Characteristics

A total of 2,516 patients with ICCA from 2010 to 2015 from the SEER database were enrolled in this study. Most of the patients with ICCA were elderly at the time of diagnosis (71.3%), white (79.2%), Non-Spanish-Hispanic-Latino (86.2%) and married or unmarried with domestic partners (60.7%). The proportions of male and female patients were approximately equal (49.9 vs. 50.1%). Most patients had tumors larger than 50 mm. More patients had moderately differentiated to poorly differentiated tumors (47.4%). More than half of the patients’ tumors were localized (stage groups I and II, 60.6%). Approximately one-third of patients had regional lymph node metastasis or distant metastasis at the time of diagnosis (30.8 and 32.4%). Most patients did not undergo any surgeries (64.5%), but more patients received adjuvant therapy alone (43.7%). In addition, most patients received chemotherapy (56.2%), while only a few received radiotherapy (16.5%). The details can be found in Table 1.

**TABLE 1:** Demographic and clinicopathological characteristics of patients with ICCA.

	Overall (*n* = 2,516)	Training (*n* = 1,762)	Testing (*n* = 754)	*p*-value
**Year of diagnosis**				
2010	300 (11.9%)	216 (12.3%)	84 (11.1%)	0.183
2011	301 (12.0%)	212 (12.0%)	89 (11.8%)	
2012	373 (14.8%)	273 (15.5%)	100 (13.3%)	
2013	417 (16.6%)	301 (17.1%)	116 (15.4%)	
2014	522 (20.7%)	345 (19.6%)	177 (23.5%)	
2015	603 (24.0%)	415 (23.6%)	188 (24.9%)	
**Age of diagnosis**				
<60 years	721 (28.7%)	490 (27.8%)	231(30.6%)	0.193
60–79 years	1,475 (58.6%)	1,037 (58.9%)	438 (58.1%)	
≥ 80 years	320 (12.7%)	235 (13.3%)	85 (11.3%)	
**Gender**				
Male	1,255 (49.9%)	870 (49.4%)	385 (51.1%)	0.465
Female	1,261 (50.1%)	892 (50.6%)	369 (48.9%)	
**Marital status**				
Single and divorced and widowed	888 (35.3%)	631 (35.8%)	257 (34.1%)	0.394
Unmarried/domestic partner and married	1,527 (60.7%)	1,056 (59.9%)	471 (62.5%)	
Unknown	101 (4.0%)	75 (4.3%)	26 (3.4%)	
**Race**				
White	1,993 (79.2%)	1,391 (78.9%)	602 (79.8%)	0.663
Black	190 (7.6%)	131 (7.4%)	59 (7.8%)	
Others	333 (13.2%)	240 (13.6%)	93 (12.3%)	
**Origin**				
Non-Spanish-Hispanic-Latino	2,169 (86.2%)	1,530 (86.8%)	639 (84.7%)	0.185
Spanish-Hispanic-Latino	347 (13.8%)	232 (13.2%)	115 (15.3%)	
**Tumor size**				
<50 mm	988 (39.3%)	698 (39.6%)	290(38.5%)	0.619
≥ 50 mm	1,528 (60.7%)	1,064 (60.4%)	464 (61.5%)	
**Grade**				
Grade I	145 (5.8%)	97 (5.5%)	48 (6.4%)	0.910
Grade II	651 (25.9%)	462 (26.2%)	189 (25.1%)	
Grade III	541 (21.5%)	378 (21.5%)	163 (21.6%)	
Grade IV	11 (0.4%)	8 (0.5%)	3 (0.4%)	
Unknown	1,168 (46.4%)	817 (46.4%)	351 (46.6%)	
**AJCC-T**				
T1	792 (31.5%)	566 (32.1%)	226 (30.0%)	0.703
T2	733 (29.1%)	508 (28.8%)	225 (29.8%)	
T3	524 (20.8%)	360 (20.4%)	164 (21.8%)	
T4	467 (18.6%)	328 (18.6%)	139 (18.4%)	
**AJCC-N**				
N0	1,741 (69.2%)	1,217 (69.1%)	524 (69.5%)	0.869
N1	775 (30.8%)	545 (30.9%)	230 (30.5%)	
**AJCC-M**				
M0	1,700 (67.6%)	1,194 (67.8%)	506 (67.1%)	0.783
M1	816 (32.4%)	568 (32.2%)	248 (32.9%)	
**AJCC-stage**				
I	535 (21.3%)	382 (21.7%)	153 (20.3%)	0.634
II	286 (11.4%)	203 (11.5%)	83 (11.0%)	
III	382 (15.2%)	256 (14.5%)	126 (16.7%)	
IVA	497 (19.8%)	353 (20.0%)	144 (19.1%)	
IVB	816 (32.4%)	568 (32.2%)	248 (32.9%)	
**Surgery**				
None	1,622 (64.5%)	/	/	/
Local destruction	80 (3.2%)	/	/	
Segmental resection	271 (10.8%)	/	/	
Lobectomy	245 (9.7%)	/	/	
Extended lobectomy	117 (4.7%)	/	/	
Hepatectomy and Transplant	154 (6.1%)	/	/	
Surgery, NOS	27 (1.1%)	/	/	
**Surgery-regional lymph nodes**				
None	1,971 (78.3%)	1,380 (78.3%)	591 (78.4%)	1.000
Yes	545 (21.7%)	382 (21.7%)	163 (21.6%)	
**Radiation**				
None and unknown	2,101 (83.5%)	/	/	/
Radiation	415 (16.5%)	/	/	
**Chemotherapy**				
None and unknown	1,103 (43.8%)	/	/	/
Chemotherapy	1,413 (56.2%)	/	/	
**Therapy**				
None and unknown	526 (20.9%)	373 (21.2%)	153 (20.3%)	0.935
Surgery alone	459 (18.2%)	324 (18.4%)	135 (17.9%)	
Adjuvant therapy	1,100 (43.7%)	765 (43.4%)	335 (44.4%)	
Systemic therapy	431 (17.1%)	300 (17.0%)	131 (17.4%)	

### Survival Analysis

**FIGURE 4:**
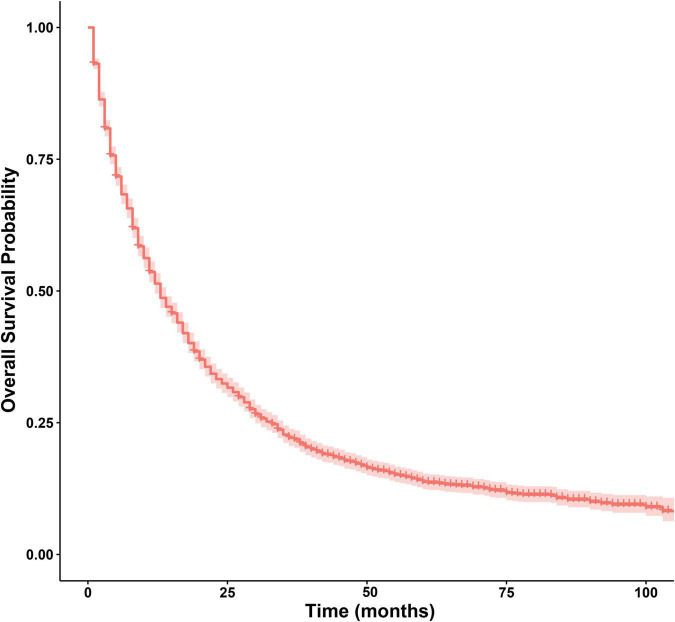
Survival curve of patients with ICCA.

In this study, 2,144 patients had died (66.5%), and 427 patients survived (33.5%). The median OS of the patients was 13 months, with a 95% CI of 12–14 months. The 1-, 3-, and 5-year OS rates were 51.40, 22.14, and 13.79%, respectively (Figure 4).

**FIGURE 5:**
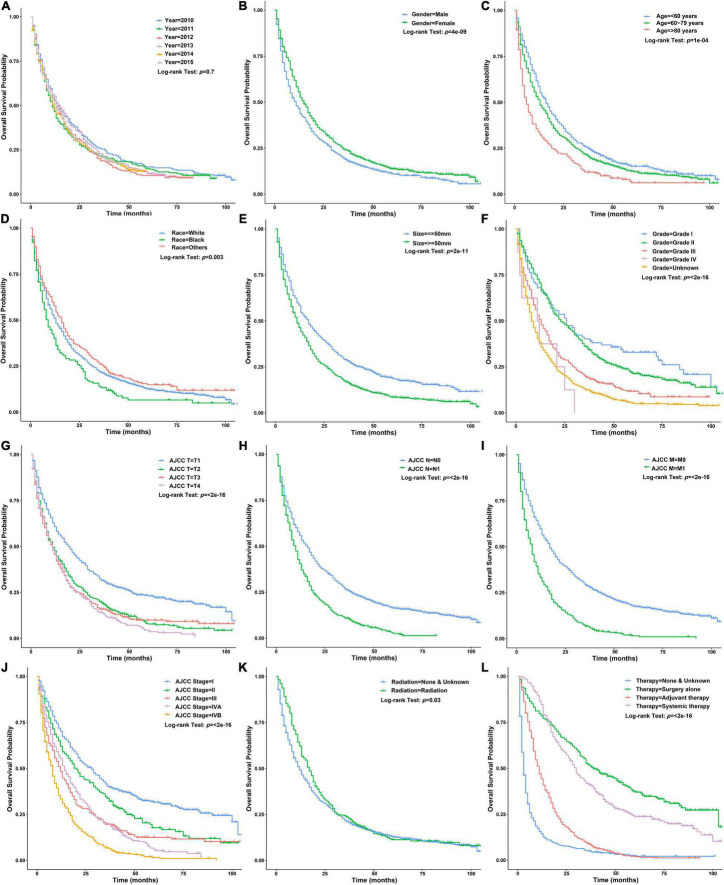
Survival curves of patients with ICCA in different clinicopathological groups in the training dataset. **(A)** Survival curves of patients with ICCA between different year of diagnosis groups. **(B)** Survival curves of patients with ICCA between different gender groups. **(C)** Survival curves of patients with ICCA between different age of diagnosis groups. **(D)** Survival curves of patients with ICCA between different race groups. **(E)** Survival curves of patients with ICCA between different tumor size groups. **(F)** Survival curves of patients with ICCA between different grade groups. **(G)** Survival curves of patients with ICCA between different AJCC-T groups. **(H)** Survival curves of patients with ICCA between different AJCC-N groups. **(I)** Survival curves of patients with ICCA between different AJCC-M groups. **(J)** Survival curves of patients with ICCA between different AJCC-stage groups. **(K)** Survival curves of patients with ICCA between different radiotherapy groups. **(L)** Survival curves of patients with ICCA between different therapy groups.

**FIGURE 6:**
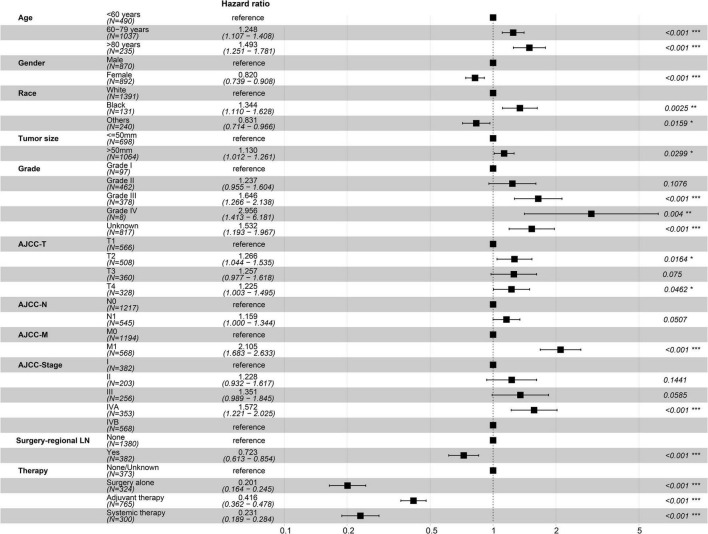
Forest map in the multivariate prognostic analysis.

There was no significant difference in baseline data between the training and testing datasets (Table 1). Univariate *Cox* regression analysis using the training dataset showed that age of diagnosis, gender, race, tumor size, grade, AJCC-T, AJCC-N, AJCC-M, AJCC-stage, regional LN surgery and therapy were related to the prognosis of patients with ICCA (Figures 5A–L and Table 2). Notably, stage IVB in the stage groups was determined by distant metastasis, meaning that stage IVB and M1 were linearly dependent covariates, thus, the above variables except AJCC-stage were enrolled in the multivariate *Cox* regression analysis. Multivariate *Cox* regression analysis showed that age of diagnosis, gender, race, tumor size, grade, AJCC-T, AJCC-N, regional LN surgery and therapy were independent prognostic factors (Figure 6 and Table 2).

**TABLE 2:** Univariate and multivariate regression analysis in the training dataset.

	Univariate analysis		Multivariate analysis	
	HR (95% CI)	*p*-value	HR (95% CI)	*p*-value
**Year of diagnosis**				
2010	1.000 [Reference]	/	/	/
2011	1.131 [0.925, 1.384]	0.230	/	/
2012	1.153 [0.953, 1.395]	0.143	/	/
2013	1.085 [0.900, 1.309]	0.392	/	/
2014	1.156 [0.962, 1.389]	0.123	/	/
2015	1.128 [0.942, 1.351]	0.189	/	/
**Age of diagnosis**				
<60 years	1.000 [Reference]	/	1.000 [Reference]	/
60–79 years	1.165 [1.036, 1.309]	0.0104	1.248 [1.107, 1.408]	3.04e-04
≥ 80 years	1.685 [1.428, 1.988]	6.6e-10	1.493 [1.251, 1.781]	8.85e-06
**Gender**				
Male	1.000 [Reference]	/	1.000 [Reference]	/
Female	0.824 [0.7448, 0.9105]	1.50e-04	0.820 [0.740, 0.908]	1.47e-04
**Marital status**				
Single and divorced and widowed	1.000 [Reference]	/	/	/
Unmarried/domestic partner and married	0.980 [0.882, 1.090]	0.714	/	/
Unknown	0.978 [0.756, 1.265]	0.864	/	/
**Race**				
White	1.000 [Reference]	/	/	/
Black	1.306 [1.081, 1.577]	0.006	1.344 [1.110, 1.628]	0.003
Others	0.885 [0.762, 1.027]	0.107	0.831 [0.714, 0.966]	0.016
**Origin**				
Non-Spanish-Hispanic-Latino	1.000 [Reference]	/	/	/
Spanish-Hispanic-Latino	1.151 [0.994, 1.334]	0.061	/	/
**Tumor size**				
<50 mm	1.000 [Reference]	/	1.000 [Reference]	/
≥ 50 mm	1.429 [1.287, 1.586]	2.13e-11	1.130 [1.012, 1.261]	0.030
**Grade**				
Grade I	1.000 [Reference]	/	1.000 [Reference]	/
Grade II	1.159 [0.897, 1.497]	0.260	1.237 [0.955, 1.604]	0.107
Grade III	1.818 [1.403, 2.356]	6.05e-06	1.646 [1.266, 2.138]	1.94e-04
Grade IV	2.792 [1.342, 5.808]	0.006	2.956 [1.413, 6.181]	0.004
Unknown	2.412 [1.886, 3.086]	2.47e-12	1.532 [1.193, 1.967]	8.18e-04
**AJCC-T**				
T1	1.000 [Reference]	/	1.000 [Reference]	/
T2	1.553 [1.361, 1.772]	6.06e-11	1.266 [1.044, 1.535]	0.016
T3	1.632 [1.412, 1.886]	3.26e-11	1.257 [0.977, 1.618]	0.075
T4	1.774 [1.532, 2.053]	1.70e-14	1.225 [1.003, 1.496]	0.046
**AJCC-N**				
N0	1.000 [Reference]	/	1.000 [Reference]	/
N1	1.653 [1.484, 1.842]	<2e-16	1.159 [0.999, 1.344]	0.051
**AJCC-M**				
M0	1.000 [Reference]	/	1.000 [Reference]	/
M1	2.153 [1.933, 2.397]	<2e-16	2.105 [1.683, 2.633]	7.14e-11
**AJCC-stage**				
I	1.000 [Reference]	/	1.000 [Reference]	/
II	1.380 [1.135, 1.677]	0.001	1.228 [0.932, 1.617]	0.144
III	1.954 [1.634, 2.337]	2.25e-13	1.351 [0.989, 1.845]	0.058
IVA	1.981 [1.681, 2.335]	3.44e-16	1.572 [1.221, 2.025]	4.57e-04
IVB	3.226 [2.774, 3.751]	<2e-16	NA	NA
**Year of diagnosis**				
**Surgery-regional lymph nodes**				
None	1.000 [Reference]	/	1.000 [Reference]	/
Yes	0.4301 [0.377, 0.491]	<2e-16	0.724 [0.613, 0.854]	1.24e-04
**Therapy**				
None and unknown	1.000 [Reference]	/	1.000 [Reference]	/
Surgery alone	0.1364 [0.114, 0.163]	<2e-16	0.201 [0.164, 0.246]	<2e-16
Adjuvant therapy	0.4776 [0.420, 0.54]	<2e-16	0.416 [0.362, 0.478]	<2e-16
Systemic therapy	0.1785 [0.151, 0.211]	<2e-16	0.232 [0.189, 0.284]	<2e-16

### Nomogram Construction

**FIGURE 7:**
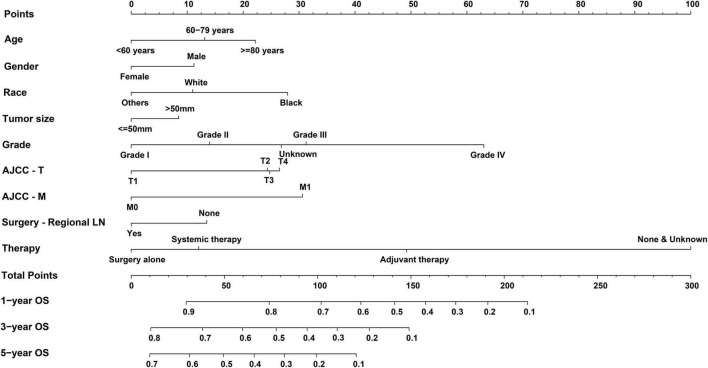
Nomogram for predicting OS of patients with ICCA.

**FIGURE 8:**
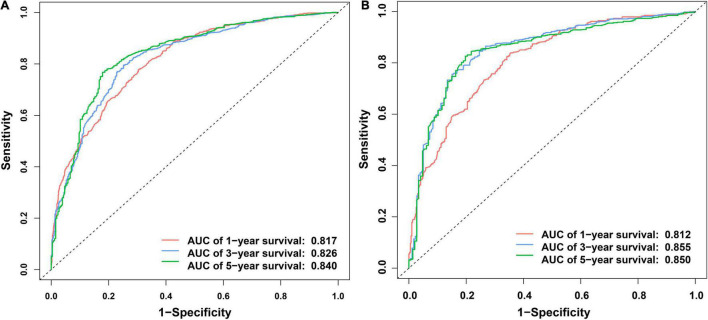
ROC curves of the nomogram. **(A)** ROC curves of the 1-, 3-, and 5- OS in the training dataset. **(B)** ROC curves of the 1-, 3-, and 5-year OS in the testing dataset.

**FIGURE 9:**
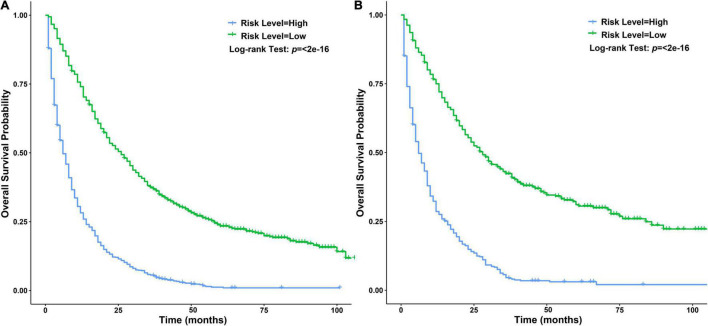
Survival curves of patients with ICCA between different risk score level groups. **(A)** Survival curves of patients with ICCA between high and low risk score level groups in the training dataset. **(B)** Survival curves of patients with ICCA between high and low risk score level groups in the testing dataset.

**FIGURE 10:**
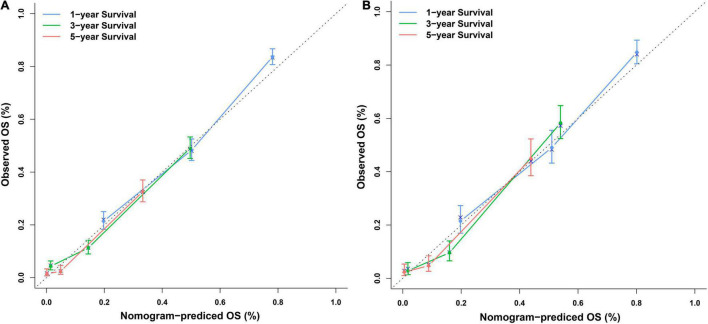
Calibration plots of the nomogram. **(A)** Calibration plots of the 1-, 3-, and 5-year OS in the training dataset. **(B)** Calibration plots of the 1-, 3-, and 5-year OS in the testing dataset.

**FIGURE 11:**
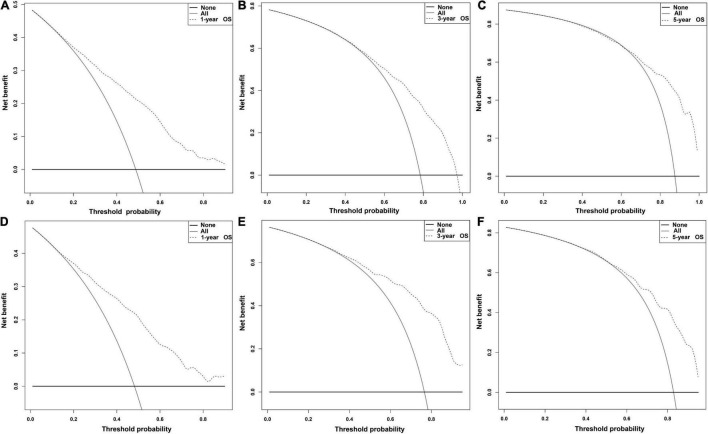
DCA plots of the nomogram. **(A–C)** DCA plots of the 1-, 3-, and 5- OS in the training dataset. **(D–F)** DCA plots of the 1-, 3-, and 5-year OS in the testing dataset.

Independent prognostic factors were used to establish a nomogram. As shown in Figure 7, patients with ICCA could get a personalized score from our nomogram, which determined the likelihood of 1-, 3-, or 5-year OS (16). In the training dataset, the AUCs of the 1-, 3-, and 5-year OS of the nomogram were 0.817, 0.826, and 0.840, respectively (Figure 8A). In the testing dataset, those values were 0.812, 0.855, and 0.850, respectively (Figure 8B). We calculated the risk scores of ICCA patients based our nomogram, and found that the prognosis of the low risk score group was significantly better than that of the high risk score group (Figures 9A,B). Therefore, the nomogram performed well in terms of discrimination. Calibration plots in the training and testing datasets showed that our curves were close to the dashed line and were roughly distributed on both sides, which indicated that the prediction by our nomogram approximated the actual outcome (Figures 10A,B) (16). DCA plots in the training and testing datasets showed that this model had better clinical utility (Figures 11A–F).

In addition, to directly benefit patients and clinical workers, we have released a free and open calculation tool for predicting the OS of patients with ICCA.^4^ On this website, users can enter patients’ clinicopathological features as needed, and then they can obtain personalized OS prediction results.

### Liver Transplantation for Patients With Intrahepatic Cholangiocarcinoma

**FIGURE 12:**
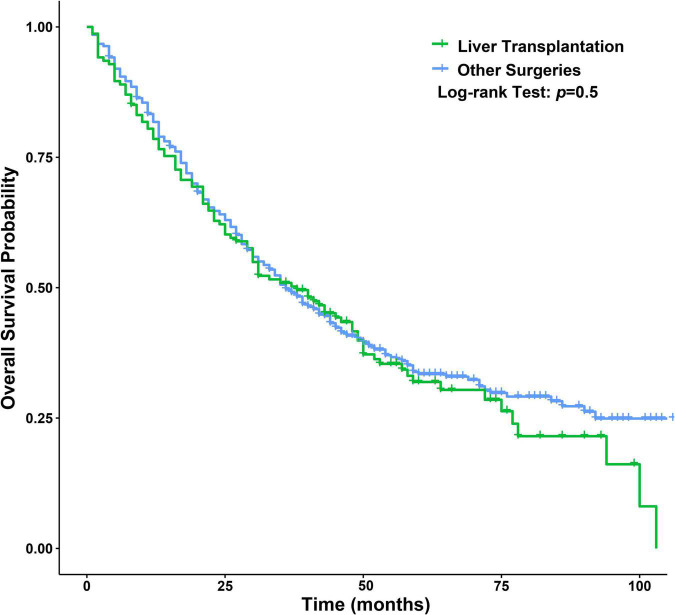
Survival curves between LT and other operations groups.

Only a small number of surgical patients underwent LT (154/787, 19.57%). As expected, we found significant differences in AJCC-T, AJCC-N, AJCC-M and AJCC stage between the two groups in the surgery dataset. After PSM, there was no significant difference in baseline data between the two groups (Table 3). The median OS of the LT group was 38 months with a 95% CI of 30–49 months, and the 1-, 3-, and 5-year OS rates were 78.52, 50.92, and 31.92%, respectively. The median OS of the other operations group was 36 months with a 95% CI of 32–42 months, and the 1-, 3-, and 5-year OS rates were 81.80, 49.70, and 33.40%, respectively. Therefore, LT and other operations could significantly improve the OS of ICCA. Univariate *Cox* regression analysis showed that there was no significant difference in the outcomes between the two groups (Figure 12).

**TABLE 3:** Comparison of demographic, clinical and treatment-related Characteristics of the surgery database before and after PSM.

	Before PSM (633 vs. 154)			After PSM (462 vs. 154)		
	Other operations	LT	*p*	Other operations	LT	*p*
**Age of diagnosis**						
<60 years	193 (30.5%)	58 (37.7%)	0.211	164 (35.5%)	58 (37.7%)	0.869
60–79 years	397 (62.7%)	88 (57.1%)		271 (58.7%)	88 (57.1%)	
≥ 80 years	43 (6.8%)	8 (5.2%)		27 (5.8%)	8 (5.2%)	
**Gender**						
Male	302 (47.7%)	79 (51.3%)	0.478	231 (50.0%)	79 (51.3%)	0.852
Female	331 (52.3%)	75 (48.7%)		231 (50.0%)	75 (48.7%)	
**Marital status**						
Single and divorced and widowed	200 (31.6%)	55 (35.7%)	0.619	152 (32.9%)	55 (35.7%)	0.811
Unmarried/domestic partner and married	407 (64.3%)	93 (60.4%)		292 (63.2%)	93 (60.4%)	
Unknown	26 (4.1%)	6 (3.9%)		18 (3.9%)	6 (3.9%)	
**Race**						
White	496 (78.4%)	132 (85.7%)	0.05	413 (89.4%)	132 (85.7%)	0.450
Black	45 (7.1%)	11 (7.1%)		26 (5.6%)	11 (7.1%)	
Others	92 (14.5%)	11 (7.1%)		23 (5.0%)	11 (7.1%)	
**Origin**						
Non-Spanish-Hispanic-Latino	559 (88.3%)	131 (85.1%)	0.336	404 (87.4%)	131 (85.1%)	0.536
Spanish-Hispanic-Latino	74 (11.7%)	23 (14.9%)		58 (12.6%)	23 (14.9%)	
**Tumor size**						
<50 mm	304 (48.0%)	83 (53.9%)	0.224	214 (46.3%)	83 (53.9%)	0.124
≥ 50 mm	329 (52.0%)	71 (46.1%)		248 (53.7%)	71 (46.1%)	
**Grade**						
Grade I	64 (10.1%)	16 (10.4%)	0.408	39 (8.4%)	16 (10.4%)	0.285
Grade II	299 (47.2%)	70 (45.5%)		210 (45.5%)	70 (45.5%)	
Grade III	176 (27.8%)	40 (26.0%)		139 (30.1%)	40 (26.0%)	
Grade IV	5 (0.8%)	4 (2.6%)		3 (0.6%)	4 (2.6%)	
Unknown	89 (14.1%)	24 (15.6%)		71 (15.4%)	24 (15.6%)	
**AJCC-T**						
T1	243 (38.4%)	45 (29.2%)	0.029	145 (31.4%)	45 (29.2%)	0.769
T2	186 (29.4%)	43 (27.9%)		138 (29.9%)	43 (27.9%)	
T3	93 (14.7%)	24 (15.6%)		72 (15.6%)	24 (15.6%)	
T4	111 (17.5%)	42 (27.3%)		107 (23.2%)	42 (27.3%)	
**AJCC-N**						
N0	498 (78.7%)	107 (69.5%)	0.02	340 (73.6%)	107 (69.5%)	0.375
N1	135 (21.3%)	47 (30.5%)		122 (26.4%)	47 (30.5%)	
**AJCC-M**						
M0	565 (89.3%)	127 (82.5%)	0.029	401 (86.8%)	127 (82.5%)	0.231
M1	68 (10.7%)	27 (17.5%)		61 (13.2%)	27 (17.5%)	
**AJCC-stage**						
I	211 (33.3%)	39 (25.3%)	0.036	119 (25.8%)	39 (25.3%)	0.722
II	125 (19.7%)	25 (16.2%)		83 (18.0%)	25 (16.2%)	
III	80 (12.6%)	17 (11.0%)		60 (13.0%)	17 (11.0%)	
IVA	149 (23.5%)	46 (29.9%)		139 (30.1%)	46 (29.9%)	
IVB	68 (10.7%)	27 (17.5%)		61 (13.2%)	27 (17.5%)	

## Discussion

Evaluating the prognosis of uncommon diseases such ICCA is difficult for both single-center and multicenter cohort studies alike. Large databases, such as the SEER database, provide new and effective tools for such research. ICCA was presented separately for the first time in the AJCC staging manual (7th edition), but some limitations with that edition were identified, so it was revised in the AJCC staging manual (8th edition); this suggests that the prognostic assessment of ICCA is still controversial (19, 20). In this study, we found that the incidence of ICCA increased significantly year by year, and we developed a nomogram with good efficacy for predicting the OS of treated or untreated patients with ICCA. By analyzing the surgery dataset after PSM, we found that LT might be a potential and effective treatment method for selected patients with ICCA, but we cannot ignore the unsatisfactory long-term OS, donor shortages and limitations of this study.

In this study, the median OS of all patients was 13 months, and the 1-, 3-, and 5-year OS rates were only 51.40, 22.14, and 13.79%, respectively. Some cohort studies showed that the 1-year OS was 83.7%, the 3-year OS was 40.8%, and the 5-year OS was 10–37.2% (10, 21–23). However, the studies mentioned above included only patients who underwent surgery, so the survival rate was somewhat different. In this study, the 1-, 3-, and 5-year OS for patients undergoing surgeries other than LT were 81.80, 49.70, and 33.40%, respectively. Therefore, the prognosis of ICCA is very poor.

The incidence of ICCA is increasing worldwide (14, 24). A population-based study showed that the incidence of ICCA increased from 0.44 per 100,000 in 1973 to 1.18 per 100,000 in 2012, with an APC of 2.3% (4). We found that the incidence rate of ICCA in the US reached 1.4% in 2017, and the incidence increased rapidly in recent years, with an APC of 7.3%. HCV, heavy drinking, obesity, and non-alcoholic fatty liver disease are the risk factors of ICCA, and *Clonorchis sinensis* is also an important cause of high prevalence of ICCA in East and Southeast Asia (1, 4, 9). In this study, we also found that the incidences of ICCA in all gender, race, age, and origin groups were increased, and males, people aged over 80 years old, people of Spanish-Hispanic- Latino descent, and native Asian and Pacific Islander populations had higher incidences than other groups. In addition, studies have confirmed that the incidence and mortality rates of ICCA in males were higher than those in females, especially in males over 45 years old (5, 24). Our results were similar two those of the studies described above, and gender was an independent prognostic factor in this study.

We found that ICCA patients with tumor size > 50 mm, lower differentiation, greater range of tumor invasion, distant metastasis, or no regional lymph node dissection had a poorer prognosis. In the AJCC staging manual (8th edition), a tumor size larger than 50 mm is one of the cutoff values for determining stages. Although it is still controversial whether tumor size affects the prognosis of ICCA patients (25, 26), tumor size is often closely related to symptoms, complications, invasion extent and treatment options. Studies have shown that tumor size might be an independent factor that affects the prognosis and recurrence of ICCA (22, 27–30). The histological grading, an important variable to be described at the pathological diagnosis reports (31, 32), was herein once again found related to the clinical behavior, with higher degrees of differentiated proved statistically related to worse prognosis. Unfortunately, since this SEER database series included cases from 1975 to 2018, the promising new histological subclassification of ICCA into large ducts/small ducts/cholangiocarcinoma subtypes could not be assessed. It is widely known that the degree of cancer cell differentiation is closely related to malignant potential and prognosis, and this was were also confirmed in ICCA patients (31, 32). Studies have shown that regional lymph node metastasis should considered an important factor for the prognosis of ICCA (21, 22, 28, 29, 33). There is no doubt about the value of regional lymph node dissection for accurate staging (11), but the therapeutic value of lymph node dissection is controversial (8, 11, 33). Studies have shown that regional lymphadenectomy might not be beneficial but also harmless to the prognosis of ICCA patients (31, 34). Some studies have shown that ICCA patients have a high incidence of regional lymph node metastasis, which may be related to postoperative recurrence and treatment plans (8, 9, 26, 27). Therefore, there was a recommendation that regional lymphadenectomy should be a standard procedure in the surgery of ICCA, which was also consistent with our findings (7, 9, 26, 35).

We found that the prognosis of the treatment group could be better, especially for patients who underwent surgery combined with adjuvant therapy. Surgery is the first choice for the treatment of ICCA, and whether margin-negative (R0) resection with sufficient FLR can be achieved is the focus of preoperative evaluation and the key to tumor-free survival (7–9, 11, 14). However, the postoperative recurrence rate and mortality rates of ICCA are high due to the advanced stage at diagnosis, the high rate of margin-positive resection and liver failure because of insufficient FLR (11, 36, 37). Therefore, preoperative biliary drainage, preoperative portal vein embolization, neoadjuvant chemotherapy and adjuvant therapy are increasingly being used in the treatment of ICCA (7, 11, 33, 38, 39). Although there is a lack of high-quality studies about the use of chemotherapy for ICCA, an increasing number of studies have shown that chemotherapy could improve the prognosis of patients with advanced staging or margin-positive resection (8, 40), and the chemotherapy is also recommended in the guidelines (7, 39).

The AJCC staging manual is the most widely used tool for predicting the prognosis of ICCA patients (19, 20), but some studies have proposed that the performance of the latest edition is unsatisfactory (41). In this study, we developed a novel nomogram to predict the OS of patients with ICCA, and this nomogram integrates predictors including age, gender, grade, tumor size, AJCC-T, AJCC-M, regional LN surgery and treatment. Treatment is often a key factor that affects prognosis. Compared with other prediction models for ICCA, we included treatment as one of the predictive factors in our nomogram, which more closely matched real-world settings, and our nomogram had better performance (30, 42–44). Our prediction model included predictors covering all patients with ICCA including untreated and treated patients, and was published online, thus our model could help to predict the prognosis and follow-up of patients with ICCA and could be more practical for use in the clinic.

Early studies revealed that patients with ICCA had an unsatisfactory prognosis after LT, so many medical centers consider ICCA to be a contraindication for LT (7, 15, 33, 45, 46). However, as the understanding of ICCA increases and LT technology improves, the application of LT for the treatment of ICCA seems to be increasing. At present, studies have indicated that LT could be used to treat patients with very early ICCA (single tumor size < 20 mm) or who meet the Milan criteria (46, 47). Studies have shown that PSC, hepatolith, liver cirrhosis and liver fibrosis are risk factors for ICCA; the incidences of positive margins, residual liver failure and intrahepatic recurrence are high after resection (1, 9, 22, 36); and multifocal tumors, positive margins and postoperative liver failure could be risk factors for prognosis after resection (7, 14). These findings seem to suggest the inevitability of LT for the treatment of ICCA because LT could eliminate potential risk factors while removing primary foci (46). Studies have found that LT combined with neoadjuvant or (and) adjuvant therapy could improve the prognosis of ICCA, especially in patients with locally advanced lesions (14, 48, 49). As we all know, LT as the last choice for the treatment of liver disease, the condition of patients receiving LT is often serious. In this study, we found that LT and other operations can improve the prognosis of patients with ICCA, and the difference between two groups was not statistically significant. Therefore, it was recommended that LT be considered an effective treatment method under strict indications, which was consistent with our results (14, 45, 46, 50, 51). However, considering the shortages of donors, and non-ideal long-term survival outcomes, we thought that LT was only a potential treatment option for ICCA according to this study.

Nonetheless, the present study had several weaknesses. First, this was a retrospective study, and most cases were excluded because of a lack of data, so selection bias may be present. Second, liver cirrhosis, PSC and other variables were not included in the SEER database, so there were limitations related to the selection of predictive factors. Third, our model was only validated internally, not externally or prospectively, which may affect its scope of application. Our goal for the future is to evaluate the performance of the nomogram through prospective external verification and further explore the indications for LT in ICCA patients.

## Conclusion

In summary, the incidence of ICCA increased significantly, and the prognosis of patients with ICCA was poor, so more attention should be paid to the management of ICCA. Furthermore, we established a nomogram with good predictive performance using prognostic factors. In some patients with ICCA for whom routine surgery is not possible, LT seems to be a potential treatment option, but the long-term outcome may be not satisfactory enough.

## Data Availability Statement

Publicly available datasets were analyzed in this study. This data can be found here: https://seer.cancer.gov/.

## Ethics Statement

The studies involving human participants were reviewed and approved by the Institutional Review Board of Children’s Hospital of Chongqing Medical University. Written informed consent for participation was not required for this study in accordance with the national legislation and the institutional requirements.

## Author Contributions

HX was responsible for conceptualization, methodology, formal analysis, and writing and editing. BT and CY were responsible for investigation and review. MZ was responsible for conceptualization, review, and supervision. All authors have read and agreed to the published version of the manuscript.

## Conflict of Interest

The authors declare that the research was conducted in the absence of any commercial or financial relationships that could be construed as a potential conflict of interest.

## Publisher’s Note

All claims expressed in this article are solely those of the authors and do not necessarily represent those of their affiliated organizations, or those of the publisher, the editors and the reviewers. Any product that may be evaluated in this article, or claim that may be made by its manufacturer, is not guaranteed or endorsed by the publisher.

## Footnotes

1
https://seer.cancer.gov

2
https://seer.cancer.gov/seerstat/

3
https://www.r-project.org

4
https://clinical-prediction-model.shinyapps.io/Prediction_Model_for_ICCA/
